# Visual Deprivation Decreases Somatic GAD65 Puncta Number on Layer 2/3 Pyramidal Neurons in Mouse Visual Cortex

**DOI:** 10.1155/2009/415135

**Published:** 2009-05-25

**Authors:** Alicja Kreczko, Anubhuthi Goel, Lihua Song, Hey-Kyoung Lee

**Affiliations:** ^1^Department of Biology, College of Chemical and Life Sciences, University of Maryland, College Park, MD 20742, USA; ^2^Neuroscience and Cognitive Science (NACS) Program, University of Maryland, College Park, MD 20742, USA

## Abstract

Proper functioning of the visual system depends on maturation of both excitatory and inhibitory synapses within the visual cortex. Considering that perisomatic inhibition is one of the key factors that control the critical period in visual cortex, it is pertinent to understand its regulation by visual experience. To do this, we developed an immunohistochemical method that allows three-dimensional (3D) analysis of the glutamic acid decarboxylase (GAD) 65-positive inhibitory terminals in the visual cortex. Using this method on transgenic mice expressing yellow fluorescence protein (YFP) in a subset of neurons, we found that the number of somatic GAD65-puncta on individual layer 2/3 pyramidal neurons is reduced when mice are dark-reared from birth and reverted to normal levels by re-exposure to light. There was no change in GAD65-puncta volume or intensity. These results support the reorganization of inhibitory circuitry within layer 2/3 of visual cortex in response to changes in visual experience.

## 1. Introduction

Proper development and function of cortical circuitry critically depends on maturation of inhibition. The majority of GABAergic inhibitory neurons are parvalbumin-positive basket cells [1], which form perisomatic synapses on excitatory neurons to exert powerful regulation of neuronal firing [2, 3]. In visual cortex, the maturation of inhibition, especially perisomatic inhibition, is regulated by visual experience [4, 5]. Throughout development, basket interneurons extend their axonal branches and form synapses all around the soma of the postsynaptic pyramidal cell, which can be visualized as GAD65-positive puncta rings [6, 7]. Previous studies have shown that visual deprivation from birth retards the developmental maturation of inhibitory circuitry [4, 8–10]. For example, dark-rearing from birth prevents the normal developmental increase in the maximal inhibitory postsynaptic current (max IPSC) onto layer 2/3 neurons of rat visual cortex [4, 8], and decreases the responsiveness to uncaged GABA at perisomatic locations [5]. Changes in inhibition are thought to underlie the increase in spontaneous action potential frequency and the degradation of receptive field properties of visual cortical neurons following dark-rearing [11]. Interestingly, the effects of visual deprivation can be readily reversed by exposure to light [4, 12] (but see [13]), and to some extent by overexpression of brain-derived neurotrophic factor (BDNF) [8]. 

The functional changes in inhibition by visual deprivation have anatomical correlates. For example, visual deprivation by intraocular tetrodotoxin (TTX) injection decreased the number of terminal boutons of parvalbumin-positive basket cells synapsing onto pyramidal neurons in layer 5/6 of mouse visual cortex [10]. While this result is consistent with a decrease in total inhibitory function by visual deprivation, there has been no direct demonstration of experience-dependent regulation of the total number of somatic inhibitory synapses, especially in layer 2/3 where the effect of dark-rearing is known to regulate inhibitory function [4, 5, 8]. Furthermore, previous studies examining GAD65 positive terminal density in layer 2/3 have reported no real change with dark-rearing [14], as well as with monocular deprivation [15], in cats. These results are inconsistent not only with the physiological findings, but also with reports of a reduction in GABAergic neurons following visual deprivation paradigms in rodents [9] as well as primates [16, 17]. This may be due to differences in species or due to limitations in the previous 2 dimensional analysis of GAD65 immunohistochemical staining. To determine anatomical changes in somatic inhibition of layer 2/3 neurons by visual deprivation, we performed a 3 dimensional analysis of somatic inhibitory synapses visualized by GAD65 immunohistochemistry. In order to quantify somatic inhibition specifically onto layer 2/3 pyramidal neurons, we used a line of transgenic mice expressing yellow fluorescence protein (YFP) in a subset of pyramidal neurons in the cortex. Here we report that the total number of GAD65-positive puncta contacting a YFP expressing soma is reduced in dark-reared mice, and is increased back to normal levels by re-exposure to 3 days of light.

## 2. Materials and Methods

### 2.1. Animals

Experiments were carried out using transgenic mice expressing yellow fluorescence protein (YFP) in a subset of layer 2/3 pyramidal neurons in visual cortex (YFP-16J line, Jackson Laboratory). Normal-reared (NR) animals were exposed to 12-hour light/12-hour dark cycles beginning at birth, and sacrificed via transcardial perfusion at postnatal day 35 (P35). In order to ensure minimal light exposure, pregnant females were placed into the dark room a few days before giving birth to the dark-reared (DR) and dark-reared followed by light exposure (LE) litters. Mice were anesthetized by halothane vapors supplied within a lightproof container and sacrificed via transcardial perfusion at P35. LE animals were kept in the dark room until P35, and then light exposed on a 12-hour dark/12-hour light schedule for 3 days before being perfused at P38. All animal procedures were carried out in accordance with the National Institute of Health Guide for the Care and Use of Laboratory Animals, and approved by the University of Maryland Institutional Animal Care and Use Committee (IACUC). Every measure was taken to minimize the number of animals used and their suffering.

### 2.2. Transcardial Perfusion

Mice were deeply anesthetized with halothane and underwent transcardial perfusion with 4% paraformaldehyde solution in sodium phosphate buffer (composition in mM: 30 mM NaH_2_PO_4_, 120 mM Na_2_HPO_4_, pH 7.4). A total of four mice were sacrificed for each experimental group (NR, DR, and LE). Brains were kept shielded from light at 4°C first in 4% paraformaldehyde solution (pH 7.4) for at least overnight up to 2 months until used for experiment. The day before sectioning, the brains were placed in 30% sucrose (in phosphate buffered saline [PBS], composition in mM: 137 NaCl, 2.7 KCl, 8 Na_2_HPO_4_, 2 KH_2_PO_4_, pH 7.4) at 4°C to sink overnight. Visual cortex was sectioned into 20 *μ*m coronal slices using a freezing sliding microtome (Leica), and stored in cryoprotectant (20% sucrose, 30% ethylene glycol, 0.02% sodium azide, in sodium phosphate buffer, pH 7.4) at –20°C away from light (less than 2 months) until antibody incubation.

### 2.3. Immunohistochemical Labeling of GAD65 Positive Terminals

At least 4 visual cortex sections from each mouse were used per incubation, and a set of experimental conditions (NR, DR and LE) was run each time. In addition, a second set of sections from the same animals were run again on a different day to control for possible variability in staining across different incubations. All incubations were done shielded from light to prevent possible bleaching of YFP. On the day of the experiment, 4 sections from each animal were removed from cryoprotectant, washed 4 times (5 minutes each) in PBS (pH 7.4), and frozen overnight at –80°C in 30% sucrose (in PBS, pH 7.4). The following day, brain sections were defrosted at room temperature and rinsed 4 times (5 minutes each) in PBS (pH 7.4). The sections were then incubated in –20°C methanol for 10 minutes, and rinsed 4 more times (5 min each) in PBS (pH 7.4). The brain sections were then incubated in blocking solution (10% normal donkey serum [NDS], 4% bovine serum albumin [BSA], and 1% Triton in PBS, pH 7.4) for 1 hour at room temperature. After blocking, the sections were incubated in GAD65 antibody (rabbit antiglutamate decarboxylase GAD65 polyclonal antibody from Chemicon) diluted 1 : 500 in blocking solution for 7 days at 4°C on a rotator. The primary antibody specificity was confirmed by a detection of a single band corresponding to GAD65 on an immunoblot (data not shown). After the 7-day primary antibody incubation, sections were rinsed in PBS (pH 7.4) 4 times (5 min each). Brain sections were then incubated for 2 hours in secondary antibody (Alexa Fluor 633 goat antirabbit IgG from Molecular Probes-Invitrogen was diluted 1 : 200 in PBS containing 1.5% NDS). The secondary antibody specificity was assessed and confirmed by no Alexa 633 signal when incubating brain sections in only secondary antibody without prior primary antibody incubation (data not shown). Sections were then mounted on superfrost VWR microslides, dried over night in the dark, and coverslipped using Prolong Gold Antifade Kit mounting solution (Molecular Probes-Invitrogen).

### 2.4. Confocal Imaging and Quantification of Fluorescence Signals

YFP expressing layer 2/3 pyramidal neurons within the visual cortex were identified and imaged using a Zeiss LSM510 confocal microscope with a 100x oil emersion objective lens. Area of imaging was selected solely on the basis of the YFP channel, hence the experimenter was blind to the GAD65 staining. Images were taken using a dual scan mode, one for YFP (emission filter: BP505–550) and the other for Alexa633 (emission filter: LP650). The confocal microscope settings for the Alexa633 channel were held constant across all the sections used in this study, and was based on a pilot experiment run to optimize the imaging conditions for GAD65 staining. The confocal microscope settings for the YFP channel were adjusted for each cell to produce saturating signals within the soma with clear cut-off boundaries. These adjustments were necessary to account for differences in YFP expression level across different cells and different animals. To obtain 3 dimensional (3D) information, 20 images were captured through the *z*-axis at 1 *μ*m steps to encompass the whole section thickness (20 *μ*m). The acquired images were analyzed using the Volocity image analysis software (Improvision), which generates a 3D image of the acquired stacks. Image stacks were acquired from at least 4 brain sections per animal per experimental group. One to three intact YFP labeled neuronal soma were selected and cropped from each image stack for a 3D analysis. Both YFP and Alexa633 signals were processed using the Volocity software by setting a threshold to eliminate background. The threshold was kept constant across each set. Only the GAD65 puncta with at least 1 voxel overlap with the YFP channel were taken as synapses contacting the YFP neuron, and the others were removed using the Volocity software. Number, volume, and fluorescence intensity of GAD65 puncta contacting each YFP labeled pyramidal cell body were then quantified. A total of 96 neuronal soma and associated GAD65 staining were analyzed from the 3 experimental groups (32 cells per group) each from 4 mice. 

For 2 dimensional (2D) analysis of images, a single section in the middle of the stack (the 10th section from the top) was used. Images with prominent blood vessels were excluded as they showed high fluorescence. Using the Volocity software, the whole field intensity of the GAD65 signal and the number of GAD65 puncta were measured. The latter was measured after thresholding to remove background staining. The threshold was kept constant for all images. GAD65 puncta density was calculated by dividing the number of puncta measured with the area of the image field.

Statistical comparison of each measurement was made using a one-way ANOVA, and *P* < .05 were taken as indicating statistically significant difference across groups. Fisher's PLSD posthoc test was run to determine further which group(s) differed from each other.

## 3. Results

### 3.1. Visual Deprivation Decreases the Number of Somatic GAD65 Puncta on Layer 2/3 Pyramidal Neurons

**Figure 1:**
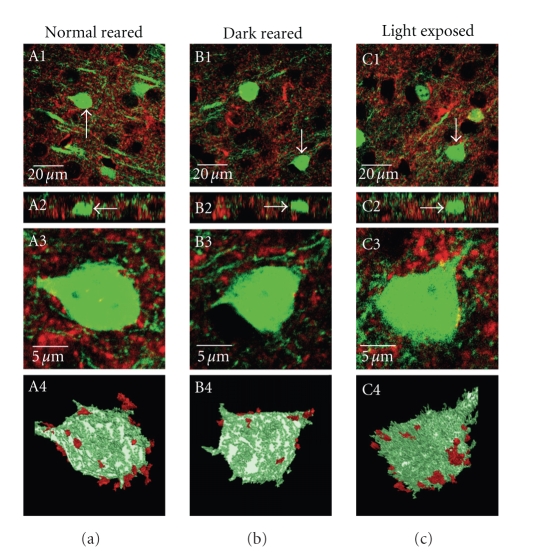
Representative examples of GAD65 immunohistochemistry in layer 2/3 of visual cortex obtained from YFP expressing mice that are reared under different experimental conditions. Confocal images obtained from mice that were normal reared for 5 weeks (A1–A4), dark reared from birth until 5 weeks old (B1–B4), and dark reared from birth for 5 weeks that were subsequently exposed to 3 days of light (C1–C4) are shown. Single section images of staining in layer 2/3 of visual cortex are shown in A1, B1, and C1. GAD65 immunoreactivity (Alexa633 conjugated secondary antibody) is shown in red, and YFP expression in a subset of pyramidal neurons is shown in green. A2, B2, and C2 show the *x*-*z* plane of each image section. Note GAD65 staining throughout the thickness of the sections. The total thickness of each section was 20 *μ*m. White arrows point to a neuron in each image field that was used for 3 dimensional (3D) reconstructions as shown in A4, B4, and C4. Single plane cropped images of the soma of selected neurons are shown in A3, B3, and C3. A stack of 20 single plane cropped sections were reconstructed to generate a 3D image using the image analysis program Volocity (Improvision). A constant threshold was applied to all images to get rid of the background in both GAD65 and YFP channels. GAD65 puncta not contacting the soma of YFP neuron were discarded using the image analysis software Volocity (Improvision) to obtain the final 3D images as shown in A4, B4, and C4. These final images were used for quantification.

**Figure 2:**
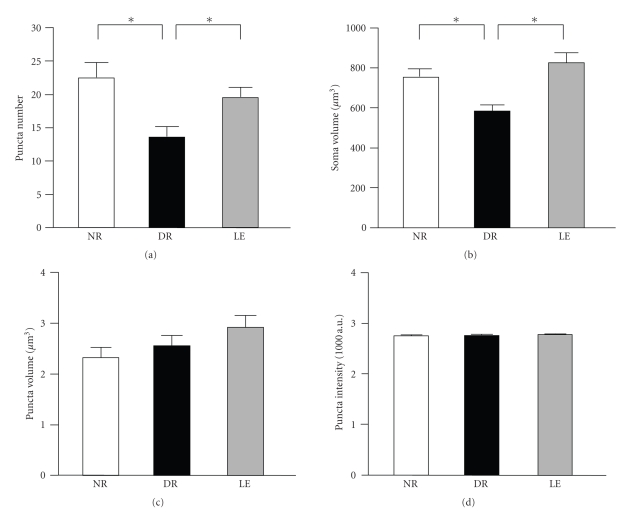
Visual experience bidirectionally changes the number of GAD65 puncta contacting the soma of pyramidal neurons in layer 2/3 of visual cortex without altering the size or intensity of GAD65 puncta. (a) Dark rearing from birth for 5 weeks (DR) significantly decreased the number of GAD65 puncta contacting neuronal soma compared to normal reared mice of equivalent age (NR). Exposing the dark reared mice to light for 3 days (LE) increased the number of GAD65 puncta close to NR levels. Asterisks indicate statistical significance of *P* < .02 using a Fisher's PLSD posthoc analysis following a one-way ANOVA. (b) The soma volume of YFP expression neurons in layer 2/3 used for measuring GAD65 puncta number. There was a statistically significant decrease in soma volume of DR mice compared to NR and LE groups. Asterisks: *P* < .01 using a Fisher's PLSD posthoc analysis following a one-way ANOVA. (c) Visual experience did not significantly alter the size of GAD65 puncta contacting the soma of layer 2/3 pyramidal cells. (d) There was no statistically significant difference in the intensity of GAD65 puncta contacting the soma of layer 2/3 pyramidal cells across the three groups.

To determine the effect of visual experience on inhibitory synaptic connections onto pyramidal neurons in layer 2/3 of primary visual cortex, we used a line of transgenic mice that express YFP in a few of these neurons (YFP16J line) and immunohistochemically labeled inhibitory terminals using an antibody against GAD65. By isolating GAD65 synaptic puncta contacting the soma of YFP expressing neurons, we were able to measure GAD65 puncta number per neuronal soma, the volume of each synaptic puncta, and the intensity of GAD65 puncta across three different conditions: normal reared (NR), dark reared from birth to 5 weeks of age (DR), and dark reared from birth followed by 3 days of light exposure (LE). A total of 4 mice were used per experimental group. One subject from each of the three groups (NR, DR, and LE) was run in parallel, and immunohistochemistry was performed in duplicate using sections from each mouse to account for possible variability across antibody incubations. Two sections from each animal per run were imaged with confocal microscopy, and on average two intact YFP labeled neurons in layer 2/3 of each section were imaged. Therefore, we imaged a total of 96 YFP expressing neurons from a total of 12 mice. Representative examples of GAD65 staining and YFP signal from a visual cortex layer 2/3 from each group is shown in Figure 1. Quantification of a total of 32 cells (from 4 mice) per group showed a significant decrease in GAD65 puncta number in dark reared mice compared to normal reared and light exposed animals (NR = 22.5 ± 2.2 GAD65 puncta/soma, DR = 13.6 ± 1.5 GAD65 puncta/soma, LE = 19.5 ± 1.5 GAD65 puncta/soma, *n* = 32 cells from 4 mice each group; one-way ANOVA: *F*(2, 93) = 6.724, *P* < .002; Figure 2(a)).

### 3.2. Visual Deprivation Decreases the Soma Size of Layer 2/3 Pyramidal Neurons

During the course of our analysis, we unexpectedly found changes in the average volume of YFP expressing layer 2/3 neuron soma by visual experience. Specifically, we found that the average soma volume in dark reared mice was significantly smaller than that of normal reared and dark reared exposed to light (NR = 758.1 ± 38.3 *μ*m^3^, DR = 584.2 ± 29.4 *μ*m^3^, LE = 824.5 ± 47.8 *μ*m^3^, *n* = 32 cells from 4 mice each group; one-way ANOVA: *F*(2, 93) = 9.880, *P* < .002; Figure 2(b)). Our measurement of the cell volume utilized YFP expression driven by a Thy-1 promoter. Differences in YFP expression levels could affect cell size measurements, because of the use of fluorescence intensity threshold for delineating the cell's boundary. However, we did not find any significant difference in average YFP intensity across the three groups (in arbitrary units of YFP fluorescence intensity: NR = 3342 ± 80, DR = 3159 ± 98, LE = 3394 ± 66; *n* = 32 cells each; ANOVA: *F*(2, 93) = 2.321, *P* > .1). While there was a trend, visual manipulation did not significantly alter the cell surface area (NR = 6774 ± 2177 *μ*m^2^, DR = 5255 ± 2007 *μ*m^2^, LE = 7318 ± 2831 *μ*m^2^, *n* = 32 cells from 4 mice each group; one-way ANOVA: *F*(2, 93) = 0.205, *P* > .8). 

The difference in GAD65 puncta number between normal reared and dark reared was not solely due to alterations in cell size, because it was still evident when puncta number was normalized to cell volume. However, the difference between dark reared and light exposure was no longer significant (NR = 0.032 ± 0.004 GAD65 puncta/*μ*m^3^, DR = 0.023 ± 0.002 GAD65 puncta/*μ*m^3^, LE = 0.024 ± 0.002 GAD65 puncta/*μ*m^3^, *n* = 32 cells from 4 mice each group; one-way ANOVA: *F*(2, 93) = 3.519, *P* < .03).

### 3.3. Visual Experience Does Not Alter the Size or Intensity of GAD65 Puncta

Next, we determined whether the different rearing conditions affect GAD65 puncta size or intensity. To do this, GAD65 puncta volume and intensity were compared across normal-reared, dark-reared, and light exposure conditions. There was no significant difference across the three conditions (Average GAD65 puncta volume: NR = 2.32 ± 0.19 *μ*m^3^, DR = 2.56 ± 0.19 *μ*m^3^, LE = 2.92 ± 0.2 *μ*m^3^, *n* = 32 cells from 4 mice each group; one-way ANOVA: *F*(2, 93) = 2.122, *P* > .1; Average GAD65 puncta intensity [measured in arbitrary units of fluorescence intensity]: NR = 2746 ± 16, DR = 2759 ± 13, LE: 2776 ± 7, *n* = 32 cells from 4 mice each group; one-way ANOVA: *F*(2, 93) = 1.424, *P* > .2; Figures 2(c), and 2(d). These results collectively suggest that visual experience bidirectionally alters GAD65 positive terminal number, but not the size or GAD65 content of individual inhibitory terminals.

## 4. Discussion

We found that depriving mice of vision for 5 weeks from birth by dark rearing decreases the number of GAD65 positive terminals contacting the soma of visual cortex layer 2/3 pyramidal neurons. The number of GAD65 puncta returned to normal levels when dark-reared mice were exposed to light for 3 days. Unexpectedly, we also found that the size of neuronal soma of layer 2/3 neurons in dark-reared mice was significantly smaller than that of normal-reared counterparts and the light exposed group. The decrease in GAD65 puncta by dark rearing is not likely a consequence of smaller soma size in the dark-reared mice, because the difference remained even when GAD65 puncta number was normalized to soma size. Interestingly, changes in GAD65 puncta number occurred without changes in the size or the average intensity of GAD65 puncta. These results suggest that dark rearing from birth decreases inhibitory input onto layer 2/3 pyramidal neurons primarily by decreasing the number of inhibitory synapses, which could be readily reversed by only 3 days of light.

### 4.1. Reversible Changes in Inhibitory Synapse Number by Visual Experience

**Figure 3:**
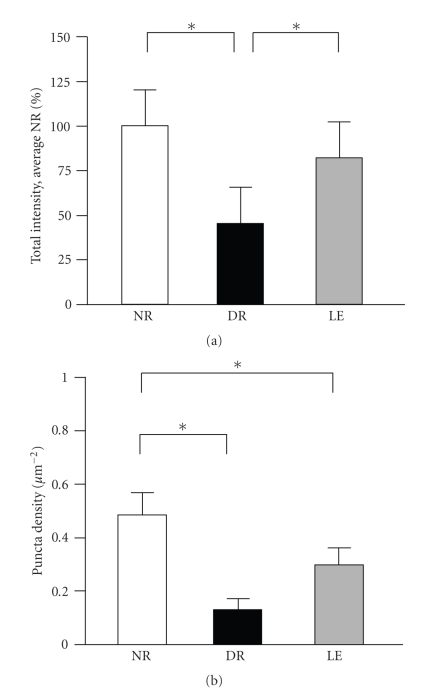
Two dimensional analysis of total GAD65 intensity and puncta density. (a) The total GAD65 staining intensity in a single focal plane was decreased in the DR group when compared to NR and LE (normalized total GAD65 intensity: NR = 100 ± 9.3%, *n* = 20 sections from 4 mice; DR = 45 ± 6.8% of average NR, *n* = 17 sections from 4 mice; LE = 82 ± 15.7% of average NR, *n* = 18 sections from 4 mice; ANOVA: *F*(2, 52) = 7.341, *P* < .01). Asterisks: *P* < .05 using Fisher's PLSD posthoc analysis. (b) The measurement of GAD65 puncta per area decreased on average in the DR group, but did not show a significant reversal with a subsequent light exposure (GAD65 puncta number per *μ*m^2^: NR = 0.48 ± 0.08, *n* = 20 sections from 4 mice; DR = 0.13 ± 0.04, *n* = 17 sections from 4 mice; LE = 0.30 ± 0.06, *n* = 18 sections from 4 mice; ANOVA: *F*(2, 52) = 6.030, *P* < .01). Asterisks: *P* < .03 using Fisher's PLSD posthoc test.

Previous results strongly suggest that dark rearing from birth attenuates the development of inhibitory networks [4, 9–11], and that exposure to light can initiate the maturation process [4, 12]. Most studies that employed GAD65 staining methods so far have relied on 2 dimensional analyses of either the GAD65 puncta number per given area [14] or average GAD65 staining intensity [15], both of which failed to detect changes with visual deprivation, or number of GAD65 puncta rings [6, 10]. We believe this is likely due to differences in either the methodology or species, because when we performed 2 dimensional analysis of our images, we saw significant decreases in both the total GAD65 intensity and the GAD65 puncta density with dark-rearing (Figure 3).

To our knowledge, our study is the first to quantify the total number of inhibitory synapses that impinge onto a single pyramidal neuron soma in an intact mouse visual cortex. Our result showing on average about 22 GAD65 positive puncta per soma of individual layer 2/3 pyramidal neuron in normal-reared mice, and about 13 GAD65 positive puncta per soma in dark-reared mice are in agreement with previous measurements. Mower and Guo (2001) reported approximately 30 GAD65 positive puncta per 400 *μ*m^2^ area in layers 2/3 of normal-reared cat visual cortex. When this information is used to calculate a theoretical number in 3D by assuming a spherical cell with a diameter of about 10 *μ*m, the result is a prediction of approximately 20 inhibitory synapses. This is in close agreement with a recent 3D analysis of somatic GAD65 puncta in organotypic slice cultures derived from mouse cortex, which measured on average about 21 [18]. Our anatomical measurements are also consistent with physiological measure of inhibition. Morales et al. (2002) quantified the maximal inhibitory postsynaptic current (max IPSC) on individual pyramidal cells within layer 2/3 of the visual cortex of rats, and also the average miniature IPSC (mIPSC), which is produced by a single inhibitory terminal bouton onto these pyramidal cells. By dividing the max IPSC value by the value of average mIPSC, the data from Morales et al. (2002) suggest approximately 21 inhibitory connections onto pyramidal cells in NR animals, and about 10 inhibitory connections onto neurons in DR animals. When these numbers are taken in context with a study exploring the number of inhibitory connections a single interneuron makes onto one pyramidal cell within layer 5/6 cells of mouse visual cortex [10], an interesting scenario is revealed. This study showed that a single parvalbumin-positive interneuron makes on average 11 inhibitory connections onto the soma of a single pyramidal cell. A similar number is reported for a basket cell making synapses onto a layer 2/3 neuron in area 18 of cat visual cortex [2]. If each interneuron creates approximately 10 synaptic contacts, and there are a total of about 20 GAD65 positive synapses on the soma of a pyramidal cell in normal-reared animals as our data indicate, this suggests that only 2 interneurons influence somatic integration in pyramidal cells. Whether layer 2/3 neurons also receive about 10 synapses from a single interneuron is unclear, but in any case this suggests that a single inhibitory neuron can have a profound effect on controlling the function of a pyramidal neuron. 

Interestingly, we show that the decrease in GAD65 puncta number by dark rearing is rather rapidly reversed by only 3 days of light exposure. This corresponds well with electrophysiological measures showing that the decrease in max IPSC by dark rearing is reversed by 2 days of light exposure [4]. Despite changes in the number of somatic GAD65 puncta, we did not find a significant change in GAD65 puncta size and intensity with visual manipulations. These results suggest that the major determinant of inhibitory strength is by regulation of the number of synaptic contacts.

### 4.2. Alterations in Neuronal Soma Size by Visual Experience

Unexpectedly, we also found a reduction in the soma size of layer 2/3 pyramidal cells in the dark-reared group, which was reversed by exposure to light for 3 days. However, we did not find a significant change in the cell surface area by visual manipulation. This suggests that the observed change in soma volume is likely due to cell shrinkage. However, we cannot make firm conclusions as the soma surface area measurements showed large variability within each group. In any case, to date there is only one other report of a reduction in cell size in visual cortex following visual deprivation. Guimarães et al. (1990) reported a reduction in cell size in cat visual cortex (area 17) by dark-rearing using a monoclonal antibody that labels putative cell surface-associated proteoglycan on a subset of neurons [19]. However, proteoglycan comprising extracellular matrix is a known target for regulation by visual experience [20], hence whether this truly reflects cell size change is unclear. The smaller soma size of layer 2/3 neurons in mice dark-reared from birth may be due to reduced availability of neurotrophins. It is known that neurotrophins can regulate cell size, which seems to be specific for distinct neuronal types. For example, NT-4 application is known to accelerate the developmental increase in soma size of lateral geniculate nucleus (LGN) neurons [21], and reverses monocular deprivation-induced cell shrinkage in the LGN [22]. On the other hand, BDNF application increases the soma size of neurons in the visual cortex [21], including layer 2/3 pyramidal neurons [23]. Dark-rearing is known to down-regulate BDNF-induced activation of TrkB receptors [24], despite increased BDNF protein expression [25]. Therefore, the smaller soma size in layer 2/3 visual cortex of dark-reared mice may reflect the reduction in BDNF signaling. The reduction in TrkB activation by dark-rearing is reported to reverse by 2 hours of light [24], hence could explain the recovery of soma size changes observed in our study. Considering that BDNF is implicated in the maturation of inhibitory synapses [6, 8, 26], it may also be responsible for the changes in GAD65 puncta number. Whether the soma size and/or the GAD65 puncta number changes observed in our study are indeed due to BDNF regulation awaits further examination.

## 5. Conclusions

We demonstrate that visual deprivation alters the number of somatic inhibitory synapses on pyramidal neurons in the superficial layers of visual cortex, which can be reversed by a brief exposure to light. Therefore, our results underscore the importance of experience in sculpting inhibitory connectivity in the cortex.
